# Recognition of depression by primary care clinicians in rural Ethiopia

**DOI:** 10.1186/s12875-017-0628-y

**Published:** 2017-04-21

**Authors:** Abebaw Fekadu, Girmay Medhin, Medhin Selamu, Tedla W. Giorgis, Crick Lund, Atalay Alem, Martin Prince, Charlotte Hanlon

**Affiliations:** 1 Center for Innovative Drug Development and Therapeutic Trials for Africa (CDT-Africa), Addis Ababa University, College of Health Sciences, PO Box 9086, Addis Ababa, Ethiopia; 2 Department of Psychiatry, Addis Ababa University, College of Health Sciences, School of Medicine, Addis Ababa, Ethiopia; 3 Department of Psychological Medicine, Centre for Affective Disorders, King’s College London, Institute of Psychiatry, Psychology and Neuroscience, London, UK; 4 Aklilu Lemma Institute of Pathobiology, Addis Ababa University, Addis Ababa, Ethiopia; 5 Federal Ministry of Health, Addis Ababa, Ethiopia; 6 Alan J Flisher Centre for Public Mental Health, Department of Psychiatry and Mental Health, University of Cape Town, Cape Town, South Africa; 7 King’s College London, Institute of Psychiatry, Psychology and Neuroscience, Health Services and Population Research Department, London, UK

**Keywords:** Detection of depression, Developing country, Ethiopia, Integrated mental healthcare, Primary care

## Abstract

**Background:**

Depression is a common health condition affecting up to a third of patients attending primary care, where most of the care for people with depression is provided. Adequate recognition of depression is the critical step in the path to effective care, particularly in low income countries. As part of the Programme for Improving Mental healthcare (PRIME), a project supporting the implementation of integrated mental healthcare in primary care, we evaluated the level of recognition of depression by clinicians working in primary care in rural Ethiopia prior to in service training. We hypothesised that the detection rate of depression will be under 10% and that detection would be affected by gender, education and severity of depression.

**Methods:**

Cross-sectional survey in eight health centres serving a population of over 160,000 people. A validated version of the 9-item patient health questionnaire (PHQ-9) was administered as an indicator of probable depression. In addition, primary care clinicians completed a clinician encounter form. Participants were consecutive primary care attendees aged 18 years and above.

**Results:**

A total of 1014 participants were assessed. Primary care clinicians diagnosed 13 attendees (1.3%) with depression. The PHQ9 prevalence of depression at a cut-off score of ten was 11.5% (*n* = 117), of whom 5% (*n* = 6/117) had received a diagnosis of depression by primary care clinicians. Attendees with higher PHQ scores and suicidality were significantly more likely to receive a diagnosis of depression by clinicians. Women (*n* = 9/13) and participants with higher educational attainment were more likely to be diagnosed with depression, albeit non-significantly. All cases diagnosed with depression by the clinicians had presented with psychological symptoms.

**Conclusion:**

Although not based on a gold standard diagnosis, over 98% of cases with PHQ-9 depression were undetected. Failure of recognition of depression may pose a serious threat to the scale up of mental healthcare in low income countries. Addressing this threat should be an urgent priority, and requires a better understanding of the nature of depression and its presentation in rural low-income primary care settings.

## Background

Depression is established as a major public health problem because of its relatively high prevalence, globally affecting 10–20% of the population [1–9], and associated disability. Depression is particularly high in primary care settings [5, 10]. In the largest study of primary care patients, which involved 14 countries, mental disorders, mostly depression, were detected, on average, in 24% of attendees [6]. The few studies from Africa suggest comparable figures [11]. Depression is the second leading cause of years of life lived with disability [12] and accounts for 11 million suicide Disability Adjusted Life Years (DALYs) and for about four million DALYS of Ischaemic Heart Disease [13]. It is often co-morbid with other medical conditions with adverse outcomes [14–16]. Depression also increases risk of mortality in high [17, 18] and low income countries alike [7, 8], even when subthreshold [19]. Although depression is treatable [20, 21], most patients with depression, particularly those from low and middle income countries (LMIC), are untreated [22, 23]. In the world mental health survey, the treatment gap for “serious cases” in “less developed” countries was 76.3–85.4% [23]. The higher treatment gap in LMIC is unlikely to be explained by scarcity of specialists alone [24]; most patients with depression in high income countries are treated by primary care doctors and not by specialists [5]. Indeed, it is estimated that up to 90% of episodes of depression are managed in primary care [25].

Integrated care, i.e., task-shifted care provided at the primary care level by non-specialists, has been the key strategy proposed to address the treatment gap in LMIC [26]. For example, the mental health gap action programme intervention guide (mhGAP-IG) of the World Health Organization (WHO) provides a set of evidence-based clinical algorithms for the assessment and management of mental disorders specifically designed for use by non-specialist health workers [26]. Yet, the primary care setting in LMIC is very different to high income settings. The most important differences are: (1) care is provided by non-physicians with lower level of training; (2) the health facility to population ratio is extremely low and therefore there is a higher level of patient flow per clinician; (3) the chronic care model in which patients have the opportunity to engage with a single physician and be monitored regularly is almost unheard of in many LMICs; (4) the profile of patients in terms of education, awareness about mental health and socio-economic status differs substantially from those in high income settings. These factors, individually and in combination, conspire against service use or detection of depression in many LMICs. Thus, whereas on average about 50% of cases with depression attending primary care in high income countries may go undetected [6, 27], studies from Africa have reported detection rates close to 0% [28, 29], which do not improve significantly with brief [29, 30] or more intensive training [28]. In the WHO supported mhGAP programme in Ethiopia, the detection of depression in the 20 pilot health centres over a 6 month period was extremely low (available at: http://www.who.int/mental_health/mhgap/mhgap_ethiopia_proof_of_concept_2013.pdf; accessed 12/02/2014). Although detection does not ensure good quality care, it is a critical step in the pathway to quality care. Poor detection will also have serious downstream impacts on efforts to scale up integrated care, including loss of political commitment. Understanding the factors that impact on detection of depression by primary care staff is of crucial importance. As part of the PRogramme for Improving Mental health carE (PRIME), which aims to support integration of mental healthcare in five low and middle income countries [31], we conducted a study to determine the detection rate of depression in the eight primary care facilities that provide care to a population of over 160,000 people [32, 33]. We hypothesised that the detection rate would be low, under 10%, and that higher education level, female gender and increased severity of depression would be associated with improved detection.

## Methods

The study was a facility-based, cross-sectional survey.

### Setting

The study was conducted in Sodo district, Gurage Zone, Southern Nations, Nationalities and Peoples Region (SNNPR), a predominantly rural district located about 100 km south of the capital city, Addis Ababa. The population of the district at the start of the project was 161,952 (79,356 men; 82,596 women) living in 58 subdistricts (*kebeles)* [32, 33]. The largest ethnic group in the district is Sodo Gurage (85.3%) followed by Oromo (11.6%) and Amhara (1.5%) and Amharic is the official language. The majority of the population is Orthodox Christian (97%), with Muslims making up 2.3%. The district has eight health centers, five rural and three urban, in addition to the health posts (total *n* = 58) found in each sub-district. The health centers are staffed by nurses and health officers (clinicians with 3–4 years of training). Health officers run outpatient clinics. Each health post is staffed by a pair of health extension workers, female high school graduates with 1 year of training in health, focusing on prevention. At the time of the study, there were no mental health services in the district other than those initiated with the support of the PRIME project. Sodo is a relatively typical rural district for Ethiopia, and is located close to the research infrastructure of the Butajira research project of severe mental disorders [8, 34] and the Butajira Demographic Surveillance Site [11, 12]. The site is also within 30–50 km distance from specialist mental health services.

### Assessment of depression and risk factors

We used the 9-item patient health questionnaire (PHQ-9) [35] to screen for depression. The PHQ was validated in the primary healthcare settings of the neighbouring district of Butajira (Meskan and Mareko) [36], as well as a general hospital setting in Ethiopia [37]. In the general hospital study, the PHQ-9 showed very good internal consistency and test-retest reliability, and at a cut-off point of 10, showed good sensitivity and specificity. However, in the primary care study, the optimum cut-off was determined to be five, with lower sensitivity and specificity. The disability item (10^th^ item) of the PHQ that enquires about impairment in day to day functioning among those who responded positively to any of the preceding nine items was also included. This item specifically asks “how difficult have these problems made it for you to do your work, take care of things at home or get along with other people?” The response options are “Not difficult at all, somewhat difficult, very difficult, extremely difficult”.

Demographic factors hypothesised to be relevant for detection (gender, education) were assessed using a structured questionnaire developed for this purpose. Severity indicators were PHQ9 score (higher score indicating more severe illness) and suicidality. Suicidality was assessed using the suicide items included in the Composite International Diagnostic Interview (CIDI) [38]. The questions in the CIDI refer to suicidal ideation, suicide plan and suicide attempt. The CIDI has good face validity, feasibility and reliability [39] and has been used previously in community based studies in Ethiopia [40, 41]. Data collectors were high school graduates with 5 years experience of data collection. They were trained for 4 days on the instruments and the instruments were then pretested on 25 adults before the conduct of the actual study.

Clinicians (health officers) working at the primary care completed a clinical encounter form. The form was a simple, custom made tool that allowed the clinician to record the presenting complaint, history of the presenting illness, any pertinent past history, findings of the physical examination, diagnosis, any investigations requested and treatment provided. In our study, all nine health officers working at the eight health centres participated. All the health officers were men. Most were aged 25 and above and had been practicing at the centres for more than 1 year. They were trained in mental health as part of their undergraduate education.

### Participants

Participants were selected through systematic random sampling of adults attending primary care facilities in all eight health centres (primary care facilities) of the Sodo district. To be included in the study, attendees had to be aged 18 years and above, be residents of the Sodo district, as evidenced by residence of 6 months or more in the district, and speak the local official language, Amharic, as the language of interview was Amharic. In estimating the sample size, we assumed a 5% detection rate at baseline (in the current study) and a 15% improvement in detection (to 20% detection) after training through the PRIME project. Assuming 80% power at 95% confidence, and 20% non-response, 978 participants were required. The eventual sample size (*n* = 1014), would enable us to estimate a detection rate by primary care clinicians of 5.0% with a margin of error of 1.3%. and a level of confidence of 95%. The number of participants for each health centre was allocated based on the number of sub-districts (anticipated population size) covered by each health centre. To obtain the number to be allocated to each health centre, the number of kebeles (nk) was divided by the total number of kebeles (NK) and multiplied by the total sample size (SS): [((nk/NK) × SS)].

### Data management and analysis

Data were entered in Epidata v3.0 (http://www.epidata.dk/) and exported into STATA for Windows (version 13) for further analysis. PHQ-9 cut-off scores of five and ten were used to distinguish severity grades of depression. Other secondary depression criteria were the PHQ-2, in which those who score 3 or more were considered to have depression [42]. The PHQ-9 was also converted into DSM-5 criteria for major depressive disorder [43]. A proportion of patients who responded positively to the PHQ items were also asked about their feelings towards discussions about stress and emotional difficulties. Furthermore, we used data from a neighbouring district, in which PHQ was validated against a gold standard diagnosis by psychiatric nurses [36], to generate weights that were then used to determine the weighted prevalence of depression. Probability of being “clinically” depressed was derived from probability weights of the Butajira PHQ validation data. In this validation study, psychiatric nurses used the Mini International Neuropsychiatric Interview with additional open clinical questions to confirm clinical depression [36]. From these validation data, the probability of receiving “valid” diagnosis of depression within a range of PHQ score categories and disability from depressive symptoms were computed. These probabilities were then used as a weight in establishing a more conservative prevalence of depression in Sodo. Analyses were primarily descriptive in nature because of the low number of participants with clinically detected depression.

## Results

### Demographic characteristics

All consecutive participants who were approached (*n* = 1014) agreed to participate and were interviewed, with complete information available for 1009 participants. Most participants were from the Gurage ethnic group (89.6%), married (63.0%) and followers of Orthodox Christian religion (Table 1). Just over half (54.1%) were women. The mean age of participants was 35.5 years (SD = 15.7) with 11.3% aged 60 or above. Nearly half of the participants had formal education (49.5%).

**Table 1:** Socio-demographic characteristics of participants ( *n* = 1014)

Characteristics		Number(%)
Sex	Male	465(45.9)
	Female	549(54.1)
Age categories (years) (*n* = 1008)	18–24	282(28.0)
	25–34	286(28.4)
	35–44	181(18.0)
	45–59	145(14.4)
	60+	114(11.3)
Age (years) (=1008)	Mean(SD)	35.5(15.7)
Marital status	Married	639(63.0)
	Never married	269(26.5)
	Formerly married	106(10.5)
Ethnicity	Gurage	908(89.6)
	Others	206(10.4)
Religion	Orthodox Christian	876(86.4)
	Protestant	105(10.4)
	Others	33(3.3)
Education	Non-literate	399(39.4)
	Literate but no formal education	113(11.1)
	Formal Education	502(49.5)
Occupation (*n* = 1012)	Civil servant	105(10.4)
	Employed in private sector	21(2.1)
	Self employed	356(35.2)
	Voluntary work	5(0.5)
	Housewife	258(25.5)
	Jobless	24(2.4)
	Student	83(8.2)
	Pensioner	20(2.0)
	Other	140(13.8)
Number of children	No child	352(34.7)
	1–4 children	346(34.1)
	More than 4 children	31.6(31.2)

### The prevalence of depression

The prevalence of depression varied depending on the cut-off score of the PHQ used to define depression (Table 2). At a PHQ9 cut-off score of 10, 11.6% of the participants had potential depression. At the lower cut-off score of five, which was the optimum cut-off in primary care settings of the region [36], the prevalence of depression was much higher, 42.8%. However, weighting back against the gold standard diagnosis from primary care data for validating the PHQ in the neighbouring district of Butajira [36], the prevalence was 7.1%.

**Table 2:** Diagnostic categories versus clinician diagnosis of study participants

Characteristics	Total n(%)	Depression n(%)	Other psychiatric morbidity (%)	Depression and other psychiatric morbidity n(%)	Epilepsy n(%)	Other health problems
PHQ9 total score						
5+	432(42.8)	13(3.0)	21(4.9)	34(7.9)	3(0.7)	395(91.4)
< 5	578(57.2)	0(0.0)	4(0.7)	4(0.7)	3(0.5)	571(98.8)
10+	117(11.6)	7(6.0)	7(6.0)	14(12.0)	2(1.7)	101(86.3)
< 10	893(88.4)	6(0.7)	18(2.0)	24(2.7)	4(0.5)	865(96.9)
PHQ2 score						
3+	116(11.5)	6(5.2)	2(1.7)	8(6.9)	3(2.6)	105(90.5)
< 3	894(88.5)	7(0.8)	23(2.6)	30(3.4)	3(0.3)	861(96.3)
DSM5 criteria						
Depressed	34(3.4)	1(2.9)	2(5.9)	3(8.8)	2(5.9)	29(85.3)
Non-depressed	976(96.6)	12(1.2)	23(2.4)	35(3.6)	4(0.4)	937(96.0)

In terms of impairment in day to day functioning, nearly 80% (79.4%) of those with PHQ9 score of five and above and over 85% of those with score of ten and above reported at least some degree of impairment.

### Clinician diagnosis of depression

In total, 38 participants (4.0%) were diagnosed by clinicians as having either depression (*n* = 13; 1.3%) or other psychiatric conditions (*n* = 25; 2.5%) (Table 3). All clinician-diagnosed cases of depression had a PHQ9 score of five and above. Most diagnosed with other psychiatric conditions (82%) also had PHQ score of at least five. Suicidality and symptom severity were associated significantly with receiving a diagnosis of depression (Table 3). Although more women and those with formal education received a diagnosis of depression, these were not statistically significant. Those receiving a PHQ2 diagnosis of depression, although not a DSM-5 diagnosis of depression, were more likely to receive a clinician diagnosis of depression (*P* = 0.007 for PHQ2).

**Table 3:** Clinician diagnosis of depression as a function of selected clinical and demographic characteristics of study participants

Characteristics	Total n(%)	Depression n(%)	Other psychiatric morbidity n(%)	Depression and other psychiatric morbidity n(%)
Severity measured with PHQ9 score				
< 5	578(57.2)	0 (0.0)	4(0.7)	4(0.7)
5–9	315 (31.2)	6(1.9)	14(4.4)	20(6.3)
10 + ^a^	117(11.6)	7(6.0)	7(6.0)	14(12.0)
Suicidality				
Yes^a^	100(9.9)	6 (6.0)	8(8.0)	14(14.0)
No	910(90.1)	7(0.8)	17(1.9)	24(2.6)
Sex				
Male	461(45.6)	4(0.9)	16(3.5)	20(4.3)
Female	549(54.4)	9(1.6)	9(1.6)	18(3.3 )
Education				
Non-literate	398(39.4)	4(1.0)	7(1.8)	11(2.8)
Literate but no formal education	124(12.3)	0(0.0)	3(2.4)	3(2.4)
Formal Education	488(48.3)	9(1.8)	15(3.1)	24(4.9)

^a^Differences statistically significant [Fisher’s exact *p* <0.001]

The sensitivity of clinician diagnosis of depression against the PHQ9 was between 2.8 and 5.1% (Table 4). This also translates into low positive and negative predictive values.

**Table 4:** Clinician diagnosis of depression and the PHQ diagnosis of depression at varied thresholds of diagnosis

Target parameter	Point estimate and confidence Interval	Detection criteria			
		PHQ9 (5+)	PHQ9 (10+)	PHQ2 (3+)	DSM 5
Prevalence	%	42.6	11.5	11.4	3.4
	95% CI	39.6, 45.7	9.6, 13.5	9.5, 13.4	2.2, 4.7
Sensitivity	%	3.0	6.0	5.2	2.9
	95% CI	1.4, 4.6	1.6, 10.3	1.1, 9.3	−3.0, 8.9
Specificity	%	100.0	99.3	99.2	98.8
	95% CI	99.4, 100.0	98.5, 99.8	98.4, 99.7	97.9, 99.4
PPV	%	100.0	53.8	46.2	7.7
	95% CI	75.3, 100.0	25.1, 80.8	19.2, 74.9	0.2, 36.0
NPV	%	58.0	89.0	89.0	96.7
	95% CI	54.8, 61.1	86.9, 90.8	86.9, 90.8	95.4, 97.7

*PHQ* Patient Health Questionnaire

### Clinical encounter

“Stress” and “headache” were the leading presenting complaints in clinically diagnosed cases of depression, occurring in 53.8% (*n* = 7/13) and 30.8% (*n* = 4/13) (Table 5), respectively. Cases with clinical diagnosis of depression had PHQ score of five and above (Table 5). “Distress” was also the most common diagnosis among attendees with a diagnosis of a psychiatric condition other than depression (Table 6). Headache was the leading presenting complaint among those with other psychiatric conditions, occurring in 57% (*n* = 16/28). Stress, dizziness and sleep problems occurred in two cases each while palpitations, chest pain, eye pain, pain during micturition, paralysis and suicidality were each presenting complaints for one case. No laboratory investigations were ordered for any of the cases diagnosed with depression. In terms of medication, eight were prescribed analgesics, two were counselled and another two were referred for further psychiatric assessment and treatment. Follow-up arrangements were made for ten of the cases. However, only five cases reported discussing about depression with the clinician. Those who discussed depression reported that they found their discussion useful (somewhat useful (*n* = 1) or very much useful (*n* = 4)).

**Table 5:** Presenting complaints and selected demographic characteristics of clinician detected cases

Age	Gender	First presenting complaint	Second presenting complaint	PHQ scores
19	F	Stress	Lack of sleep	15
19	M	Stress	--------	8
20	F	Headache	Fever	10
20	F	Stress	--------	7
20	F	Stress	--------	5
20	F	Headache	Depression	14
23	M	Suicide attempt	Sleep problem	10
25	F	Headache	Hearing difficulty	16
25	M	Stress	Lack of sleep	6
38	M	Depression	Headache	12
40	F	Fever/Headache	Ear discharge	9
41	F	Stress	-------------	5
44	F	Stress	Headache	10

**Table 6:** Psychiatric diagnosis other than depression made by primary care clinicians

Specific diagnosis	Number	Percent
Schizophrenia	1	3.6
Anxiety disorder	4	0.4
Distress	12	1.2
Insomnia	2	0.2
Mental and behavioural disorder	5	0.5
Worry (“Thinking”)	1	0.1
Suicidality	1	0.1
Total	26	2.6

## Discussion

Depressive symptoms are prevalent in this sample of primary care attendees. While nearly half of the participants had depressive symptoms above the optimum cut-off point of five, about 11% had symptom severity compatible with a diagnosis of major depressive disorder (PHQ score of 10 and above). Yet, the detection rate of depression by primary care clinicians was extremely low, with over 95% of patients presenting to primary care with potential depression not receiving a clinical diagnosis of depression. This level of non-detection is much higher than reported in high income countries where about 50% of cases with depression may be unrecognised [44]. With ongoing engagement for a period of 6 months, the proportion with unrecognized depression goes down to 20% [45]. However, such an alarmingly low rate of detection is not unique for these rural Ethiopian facilities with the limited evidence from sub-Saharan Africa indicating a detection rate of depression in primary care close to 0% [11, 28]. The significance of this finding looms large in the context of the current international effort to scale up mental health care in low and middle income countries through services provided by primary care clinicians. It is also very important because most patients with depression, whether in low income or high income country settings, are bound to receive treatment in primary care. Thus the low detection represents a major threat as well as opportunity to address an important public health challenge.

Although further work is required, the apparent low detection of depression is unlikely to be due to low rates of depression. In a validation study in primary care facilities of the adjoining rural districts of Meskan and Mareko, the prevalence of depression based on mental health professional diagnosis was 5% [36]. In the current study, nearly half of the participants had depressive symptoms with a PHQ score of five and above while about one in ten had a PHQ score of ten and above. Although not all those with high levels of PHQ depressive symptoms are likely to have major depressive disorder, it appears that these symptoms might not have been trivial in that the majority of participants who reported depressive symptoms also reported at least some degree of impairment in their day to day functioning. Suicidal ideation was also increased among those reporting depressive symptoms in the PHQ rating. These provide indirect evidence for the clinical relevance of the depressive symptoms recorded. Although studies from high income countries suggest that those with undetected depression have mild illness and benefit of treatment may be uncertain [41], the extremely low detection rate in the current study and in the few studies from Sub-Saharan African countries mean that a proportion of the missed cases are likely to have more severe illness and to benefit from treatment.

The reasons for the low detection are likely to be multifaceted and related to health system features, and patient and clinician factors [46–49]. The health system in many low and middle income countries, particularly in sub-Saharan African countries, is geared towards acute care. Although, ongoing contact with a service increases the chances of detection, patients in these settings are rarely seen more than once and clinicians and patients rarely form ongoing therapeutic relations. Explanatory models of illness regarding mental illness, which tend to be shared by clinician and patient with mental disorders, are likely to influence both clinician and patient. Social attributions, emotional burden and concern about dealing with social issues that might have led to the depression in the first place have been reported as important barriers [48]. The expectation of patients, and how symptoms are presented and communicated influence the ability of the clinician to detect depression. Family members are also important in influencing decisions of diagnosis and treatment and their explanatory model is likely to have a role. Clinicians and patients are also likely to share similar attitude towards mental illness as the community they live in. Negative attitude towards mental illness is thus likely to influence the diagnosis of depression. The low level of knowledge and skill of clinicians have important impact on diagnosis. Recognition of depression may be affected by a number of illness related features. Presentation with mood symptoms, past history of illness, more severe depressive symptoms and reporting suicidal tendencies increase the chance of detection [50]. In the current study, clinician detection of depression relied on direct report by patients of psychological symptoms; however, in primary care settings, most patients present with somatic rather than psychological symptoms [5]. Improving detection of depression in routine care requires interventions that address the system level needs, and the needs of the clinician as well as addressing patient and family/community level barriers.

Detection does not always lead to care. For example, a series of studies conducted in high income countries have indicated that only a quarter to one third of those detected receive appropriate intervention [51–53]. Therefore, intervention to improve detection should also address this “detection-intervention” gap. Addressing this gap will require changes at all the levels that are relevant for detection. The health system need to adopt the chronic care approach, which has the potential not only to improve depression care but also cost [54]; clinicians have to be equipped with the necessary knowledge and skill; the attitude of clinicians, patients and the public at large needs to encourage detection and treatment.

The results of this study should be interpreted with some important limitations in mind. The proportion with detected depression was too small to make generalizable conclusions regarding the implication of the detected depression. The report is also primarily descriptive because of the apparent risk of unstable estimation. However, although this is an important limitation, we believe the descriptive nature of the paper should not detract from the clear relevance of its content. First, the paper fills an important knowledge gap regarding the detection of depression in LMICs given the dearth of evidence in LMIC settings. Secondly, as we have indicated, our report comes in the context of the concerted effort to scale up mental healthcare in LMICs. In this regard, the report shows a crucial challenge for scaling up care and has the potential to spur researchers and clinicians to develop interventions to improve detection of depression. Detection is an unresolved conundrum that awaits concerted multidisciplinary effort, particularly in LMICs. Thirdly, despite the limited number, we have provided detailed clinical description of the cases, which might be used for training of primary healthcare workers.

Further assessment to understand what the PHQ symptoms mean would have allowed a more accurate interpretation of the ratings. The reasons provided for the under-detection are theoretical and further studies to understand the role of the various proposed mechanisms for the low detection, for example through qualitative studies, need to be explored. The cross-sectional nature of the assessment may introduce bias, particularly recall bias. However, the depressive symptoms were assessed for the preceding 2 weeks and the impact of recall bias should be minimal.

## Conclusion

Detection is the rate limiting step in the path to care for people with depression in low and middle income countries. Findings from this study indicate that the level of detection is unacceptably low. Even if it was concluded that only a small proportion of these cases would require treatment, still a major opportunity for intervention has been missed.

However, several issues have to be addressed in order to deal with the detection barrier. First, understanding the question of the nature of depression in these settings is important and should be explored carefully. The second crucial issue is understanding the process of detection. Finally understanding the system level barriers and the organizational or structural requirements of the health system are crucial. Improving detection should be one of the priorities of global mental health studies.

## Abbreviations

CIDI: Composite International Diagnostic Interview
DALY: Disability adjusted life year
LMIC: Low and Middle Income Countries
mhGAP: Mental health Gap Action Programme
mhGAP-IG: mhGAP intervention guide
PHQ: Patient Health Questionnaire
PRIME: Programme for Improving Mental Health Care
SNNPR: Southern Nations Nationalities and Peoples Region
WHO: World Health Organization
